# Chronic Ethanol Consumption in Rats Produces Opioid Antinociceptive Tolerance through Inhibition of Mu Opioid Receptor Endocytosis

**DOI:** 10.1371/journal.pone.0019372

**Published:** 2011-05-13

**Authors:** Li He, Jennifer L. Whistler

**Affiliations:** 1 Ernest Gallo Clinic and Research Center, University of California, Emeryville, California, United States of America; 2 Department of Neurology, University of California, San Francisco, California, United States of America; Tokyo Institute of Psychiatry, Japan

## Abstract

It is well known that the mu-opioid receptor (MOR) plays an important role in the rewarding properties of ethanol. However, it is less clear how chronic ethanol consumption affects MOR signaling. Here, we demonstrate that rats with prolonged voluntary ethanol consumption develop antinociceptive tolerance to opioids. Signaling through the MOR is controlled at many levels, including via the process of endocytosis. Importantly, agonists at the MOR that promote receptor endocytosis, such as the endogenous peptides enkephalin and β-endorphin, show a reduced propensity to promote antinociceptive tolerance than do agonists, like morphine, which do not promote receptor endocytosis. These observations led us to examine whether chronic ethanol consumption produced opioid tolerance by interfering with MOR endocytosis. Indeed, here we show that chronic ethanol consumption inhibits the endocytosis of MOR in response to opioid peptide. This loss of endocytosis was accompanied by a dramatic decrease in G protein coupled receptor kinase 2 (GRK2) protein levels after chronic drinking, suggesting that loss of this component of the trafficking machinery could be a mechanism by which endocytosis is lost. We also found that MOR coupling to G-protein was decreased in ethanol-drinking rats, providing a functional explanation for loss of opioid antinociception. Together, these results suggest that chronic ethanol drinking alters the ability of MOR to endocytose in response to opioid peptides, and consequently, promotes tolerance to the effects of opioids.

## Introduction

The mu opioid receptor (MOR) has long been proposed to contribute to alcohol consumption [1], [2]. Indeed, blocking the MOR with an opioid antagonist significantly reduces ethanol consumption in both animal models (see for example [3]) and, with variable efficacy, in humans [4]–[7]. In further support of a role for MOR in drinking, animals with higher levels of MOR in certain brain regions drink more alcohol compared to animals with lower MOR levels [8], [9]. Moreover, animals with an RNAi knock-down of MOR [10], or a genetic disruption of MOR, show reduced drinking and reward to ethanol [11], [12]. Taken together, these studies strongly implicate the MOR in the mechanisms underlying alcohol consumption and, potentially, its abuse.

Despite the clear role of MOR in modulating ethanol consumption and reward, few studies have examined the effects of drinking on signal transduction from the MOR. Importantly, ethanol has been shown to increase release of endogenous opioids in brain regions key to the rewarding properties of drugs of abuse [13]–[15]. This would be expected to increase signal transduction through MOR, in effect signaling the presence of ethanol. By extension, opioid antagonists, such as naltrexone, are thought to decrease ethanol consumption because they block the actions of these endogenously released opioids. On the other hand, chronic ethanol consumption appears to decrease the functional responsiveness of the MOR [16]–[18], suggesting that adaptations can occur during chronic ethanol exposure that make the MOR less sensitive to the same dose of opioid. However, the mechanisms that mediate these decreases in the sensitivity of MOR responses are not known.

After activation by endogenous opioids, signaling from the MOR is regulated by many processes, including desensitization by G protein coupled receptor kinases (GRK) and arrestins, endocytosis of the receptor [19], and recycling and resensitization of the receptor [20]. This cascade of events serves to carefully titrate signal transduction from the receptor and is ideally suited to monitor the presence of ligands, such as the endogenous opioid peptides that are released in a pulsatile manner. Agonist ligands such as morphine that do not promote desensitization/endocytosis/resensitization of the MOR, have been shown to facilitate homeostatic adaptive responses in signal transduction that manifest as reduced responsiveness to the presence of opioid ligands at the cellular level, and as antinociceptive tolerance at the behavioral level (for review see [21]). Here, we found that chronic voluntary consumption of ethanol causes a downregulation of GRK and prevents endocytosis of the MOR in response to opioid peptide ligand. As a consequence, rats consuming ethanol show tolerance to the antinociceptive effects of opioids.

## Materials and Methods

### Ethics statement

All experiments with vertebrate animals were approved by the Institutional Animal Care and Use Committee at Ernest Gallo Clinic and Research Center, University of California, San Francisco, protocol number 08.06.173 and were in accordance with National Institutes of Health Guide for the Care and Use of Laboratory Animals.

### Animals and drugs

Male Wistar rats (200–220 g) were purchased from Charles River Laboratories (Wilmington, MA) and housed individually in a temperature controlled environment (22±2°C) with a 12-hour light/dark cycle. Rats were given 1 week to acclimatize to the new housing conditions with food and water available ad libitum before being entered into the study. All experiments with vertebrate animals were approved by the Institutional Animal Care and Use Committee at Ernest Gallo Clinic and Research Center, University of California, San Francisco, and were in accordance with National Institutes of Health Guide for the Care and Use of Laboratory Animals. [^3^H]–Ala^2^-MePhe^4^-Gly-ol^5^ enkephalin (DAMGO) (50 Ci/mmol) was purchased from PerkinElmer Life Science (Boston, MA, USA). Morphine sulfate was obtained from Sigma Chemical (St. Louis, MO, USA). DAMGO was purchased from Tocris Cookson, Inc (UK). The drugs were dissolved in physiological saline and injected intrathecally (i.t.) in a volume of 5 µl/rat as previously described [22], [23].

### Ethanol Drinking Procedure

The intermittent access 20% ethanol two-bottle choice drinking paradigm was used as previously described with minor modifications [24]. In brief, each week rats were given 24 hour access to ethanol (20% v/v) and water in 100-ml graduated glass cylinders with stainless-steel drinking spouts on Monday, Wednesday, and Friday. On Tuesday and Thursday the ethanol bottle was replaced with a second water bottle that was available for 24 hours. The rats had unlimited access to water over the weekend after the 24-hour ethanol drinking measurements were taken on Saturday morning. Bottles were weighed to the nearest gram.

### Antinociception assessment

Drugs were administered i.t. in a volume of 5 µl/rat as previously described [22], [23].

Antinociception was tested using the radiant heat tail-flick procedure 30 minutes after i.t. drug administration. The light intensity was adjusted to achieve baseline (drug free) latencies of 3 to 4 seconds and a maximum latency of 10 seconds was set as the cut-off time to minimize damage to the tail. Data are reported as maximal possible effect (MPE), calculated as 100% × [(drug response time - basal response time)/(cutoff time - basal response time)].

### Immunohistochemistry

Rats were anesthetized with isofluorane and perfused with 4% paraformaldehyde in 0.1 M phosphate buffer immediately following the tail-flick test. The lumber segment of the spinal cord was dissected out, post-fixed overnight, and then transferred to a 30% sucrose buffer solution. Sagittal sections (30 µm) were cut on a cryostat at −20°C, pre-incubated in PBT solution (0.1 M phosphate buffer, 0.2% BSA, 0.2% Triton X-100) for 30 minutes, blocked in 5% normal goat serum in PBT solution for another 30 minutes, and then incubated overnight at 4°C with rabbit anti-MOR antibody (1∶2000, ImmunoStar, Inc, Hudson, WI, USA) and either mouse anti-NeuN antibody (1∶5000, Chemicon International, Temecula, CA, USA) or mouse anti-Rab5 (1∶1000, Abcam Inc., Cambridge, MA, USA). Sections were then extensively washed with PBT and incubated for 2 hours at room temperature with Alexa 488-conjugated goat anti-rabbit antibody and Alexa 546-conjugated goat anti-mouse antibody (Invitrogen, Carlsbad, CA, USA), both at 2 µg/ml. The sections were then washed and mounted on slides. MOR distribution was examined with a Zeiss confocal microscope using a 63× oil immersion objective. The intracellular MOR vesicles were counted using ImageJ software by an investigator blind to treatment. The plasma membrane was first selected as the border of the measurement. The threshold and the size of vesicles to be measured were equal among the different treatment groups. Ninety neurons taken from 2 to 5 rats per group were quantified.

### GRK2 Immunoblot

Rats were sacrificed by decapitation after 4 or 8 weeks of water or ethanol drinking, and the lumber segments of spinal cord were quickly dissected out on ice. Tissue was homogenized in ice-cold buffer [10 mM Tris-HCl and 5 mM EDTA (pH 7.4)] containing a protease inhibitor cocktail (Roche Diagnostics, Indianapolis, IN). The homogenates were stored at −80°C until use. Proteins were solubilized by boiling in 2% SDS containing 50 mM dithiothreitol, resolved by 4–12% SDS-PAGE (15 µg/lane) and transferred to PVDF membranes. The membranes were incubated in blocking buffer (5% nonfat dry milk in 20 mM Tris-HCl containing 0.1% Tween-20, 140 mM NaCl pH 7.4) for 1 h at room temperature and then incubated in blocking buffer with monoclonal anti-GRK2 antibody (1∶5,000, Santa Cruz Technology, Inc., Santa Cruz, CA, USA), and monoclonal anti-β actin antibody (1∶10,000, Sigma, St. Louis, MO) overnight at 4°C. After extensive washing with Tris-buffered saline containing 0.1% Tween-20, the membranes were incubated with horseradish peroxidase-conjugated goat anti-mouse antibody at a dilution of 1∶10,000 for the GRK2 and 1∶20,000 for the β actin for 1 h at room temperature in blocking buffer. Following several washes with Tris-buffered saline containing 0.1% Tween-20, proteins were visualized with enhanced chemiluminescence. Densitometric quantification of immunoreactive bands was performed using Scion Image software (Scion Corporation, Frederick, MD USA). The relative GRK2 protein levels were obtained by normalizing the anti-β-actin immunoblot against the corresponding GRK2 immunoblot from the same membrane and are expressed as a percentage relative to the control group. The differences between control and ethanol drinking groups were analyzed by a Student *t* test.

### Tissue section preparation for autoradiography

Rats were sacrificed by decapitation. Brain and the lumber segment of spinal cord were quickly dissected out on ice and were frozen in isopentane on dry ice and stored at −80°C. Coronal sections (16 µm) were cut on a cryostat at −20°C and thaw-mounted onto gelatin-subbed slides. The slides were dried and stored desiccated at −80°C until use.

### [^3^H]DAMGO autoradiography

Sections (prepared as above) were preincubated in assay buffer (50 mmol/l Tris- HCl, pH 7.4) for 30 minutes and then incubated in the same buffer containing [^3^H]DAMGO (4 nM) for 60 min at room temperature as previously described [25]. Nonspecific binding was defined as the [^3^H]DAMGO signal obtained in the presence of 1 µM of unlabeled naloxone on the adjacent sections. After incubation, slides were rinsed twice in cold 50 mM Tris-HCl buffer, pH 7.4, and once in deionized water, dried and exposed to Kodak BioMax light film with an intensifying screen for 3 weeks. Slides containing PAG sections were exposed for 4 weeks. The films were developed using a medical film processor (SRX-101A, Konica Minocta). At least 4 sections from a minimum of 3 animals per group were quantified.

### DAMGO-activated [^35^S]GTPγS autoradiography

Sections were pre-incubated in assay buffer (50 mM Tris-HCl, 3 mM MgCl, 0.2 mM EGTA, 100 mM NaCl, pH 7.4) at room temperature for 10 minutes and then incubated with 2 mM GDP in assay buffer for 10 minutes at room temperature as previously described [26]. Sections were then incubated for 90 min at room temperature in assay buffer containing [^35^S]GTPγS (0.05 nM) and 2 mM GDP, with and without the agonist DAMGO (2 µM). After incubation, slides were rinsed twice in cold 50 mM Tris-HCl buffer, pH 7.4, and once in deionized water, dried, and exposed to Kodak BioMax light film for 3 days. At least 4 sections from a minimum of 3 animals per group were quantified.

### Statistics and image analysis

The antinociceptive effects of the drugs between treatment days or between water and ethanol drinking rats were analyzed by a Student *t* test. The regional neuroanatomy of the rat was determined using the atlas of Paxinos and Watson [27]. Brain areas in the autoradiograms and spinal cord were quantified using Scion Image software (Scion Corporation, Frederick, MD, USA). For [^3^H]DAMGO autoradiography, optical densities were converted into fmol/mg tissue for quantification of MOR sites according to a [^3^H] microscale standard (GE Healthcare UK Limited). For DAMGO-activated [^35^S]GTPγS autoradiography, data were expressed as % activation, where % activation  =  [(activated – basal)/basal] X 100. The data are expressed as mean ± S.E.M. and were compared using a Student *t* test. The comparisons of intracellular vesicle counts among groups were analyzed with a one-way ANOVA followed by a Newman-Keuls post-test. Results were considered significant if p≤0.05. All statistical analyses were done using GraphPad Prism Software (San Diego, CA, USA).

## Results

### Ethanol drinking induces tolerance to exogenous opioids

We and others have reported that ligands that promote endocytosis of the MOR produce reduced antinociceptive tolerance compared to morphine, which does not promote MOR endocytosis. These results have led us to hypothesize that endocytosis protects against the development of tolerance. To further examine this hypothesis, we tested the ability of equi-antinociceptive morphine and DAMGO, a hydrolysis-resistant form of one of the endogenous ligands at the MOR, to produce antinociceptive tolerance.

Rats were injected with intrathecal (i.t.) morphine or DAMGO twice daily for 4 days and were tested on days 1 and 5 for their antinociceptive response to these two drugs. Rats developed significant antinociceptive tolerance to morphine, while DAMGO remained as effective on day 5 as day 1 (Figure S1A).

**Figure 1:**
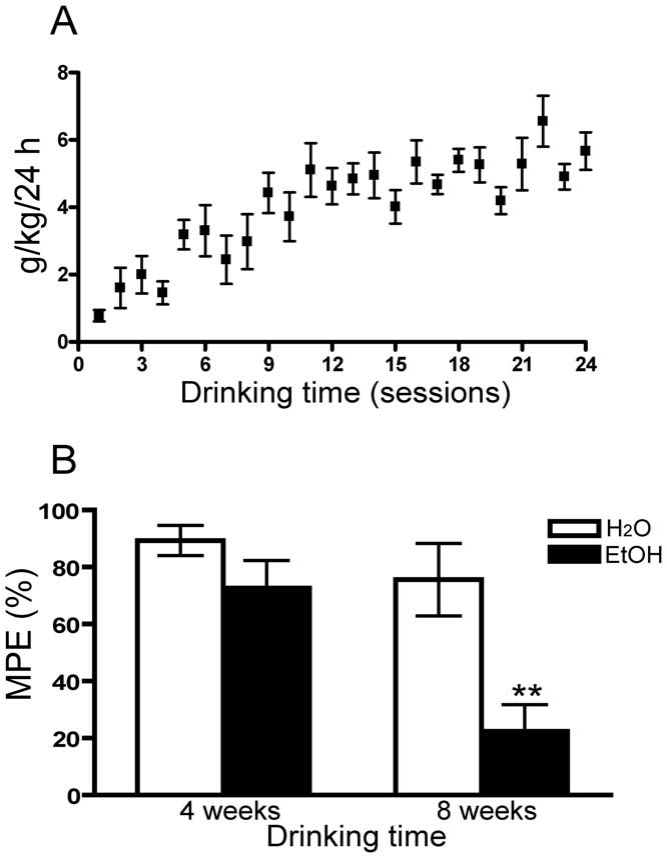
The antinociceptive effects of intrathecal (i.t.) DAMGO following chronic ethanol consumption. (A) Rats were given intermittent access to 20% ethanol (three 24-hour sessions per week). Ethanol intake escalated over the first 5 to 6 drinking sessions, and rats reached a stable consumption of ethanol by session 14. (N = 30) (B) The antinociceptive effect of DAMGO (1 nmole, i.t.) was tested after 4 and 8 weeks of ethanol or water only drinking. After 8 weeks of ethanol consumption, rats were tolerant to the antinociceptive effects of DAMGO (N = 8 for both water and ethanol drinking groups. **p<0.01; ethanol vs. water drinking group).

We next examined whether chronic ethanol drinking would alter the antinociception produced by opioid drugs. Rats were trained to drink ethanol in an intermittent-access paradigm [24] in which they were given access to 20% ethanol for three 24-hour sessions per week. During the first 5 to 6 drinking sessions rats escalated their consumption, reaching a stable level of drinking by session 14 (Figure 1A). The antinociceptive effects of the opioid peptide DAMGO (i.t, 1 nmol/rat) was assessed at two time points: 4 and 8 weeks after initiation of alcohol drinking. After 8 weeks of drinking, rats had developed significant antinociceptive tolerance to DAMGO (Figure 1B). Not all paradigms that produce antinociceptive tolerance produce cross tolerance to multiple opioid drugs [28]–[31]. However, we found that chronic voluntary ethanol consumption produced tolerance to not only DAMGO but also morphine (i.t. 20 nmol/rat, Figure S1B). These decreases in antinociception were not due simply to increased age, since water drinking animals did not develop antinociceptive tolerance to opioid drug (Figure 1B, S1B). Ethanol itself can produce antinociceptive effects [32], [33], at least some of which are mediated by the MOR [34]. However, a direct effect of ethanol on DAMGO-mediated antinociception in these drinking rats is unlikely since DAMGO antinociception was tested 24 hours after their last ethanol exposure, and blood alcohol concentration at that time was no different than that in water drinking group.

### The effects of chronic ethanol drinking on MOR levels

**Figure 2:**
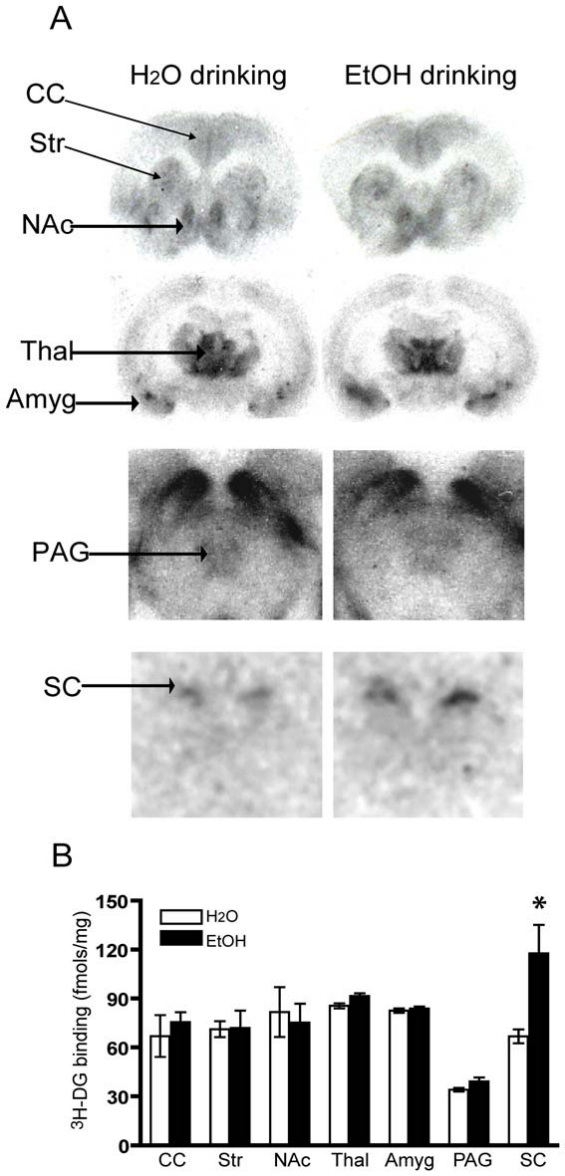
Comparison of MOR density in the CNS after 8 weeks of drinking. (A) Representative autoradiograms showing [^3^H]DAMGO binding in coronal sections from water and ethanol drinking rats. (B) Histograms show the quantification of MOR binding from different CNS regions of ethanol and water drinking rats. Results are expressed as the mean ± SEM value from 3–4 rats in each group with 4–6 sections for each rat. * p<0.05, compared with water drinking group. CC  =  Cingulate cortex; Str  =  striatum; NAc  =  nucleus accumbens; Thal.  =  thalamus; Amyg.  =  amygdala; PAG  =  periaqueductal gray; SC  =  spinal cord.

It was possible that the reduction in the antinociceptive effect of i.t. DAMGO was a consequence of MOR down-regulation. To examine this possibility, we analyzed MOR density in water and ethanol drinking rats. [^3^H]DAMGO binding in the spinal cord and several brain regions was assessed. There was no difference in receptor density between the water and ethanol drinking rats in any of the brain regions examined (Figure 2A, B). However, somewhat surprisingly, we observed an increase rather than a decrease in MOR number in the spinal cord of ethanol drinking rats relative to the water drinking group despite the fact that i.t. DAMGO produced less antinociception in the ethanol drinking rats (Figure 1). Hence, it is unlikely that tolerance to the antinociceptive effects of opioids after chronic drinking is due to downregulation of MOR.

### Ethanol drinking alters the trafficking of the MOR

**Figure 3:**
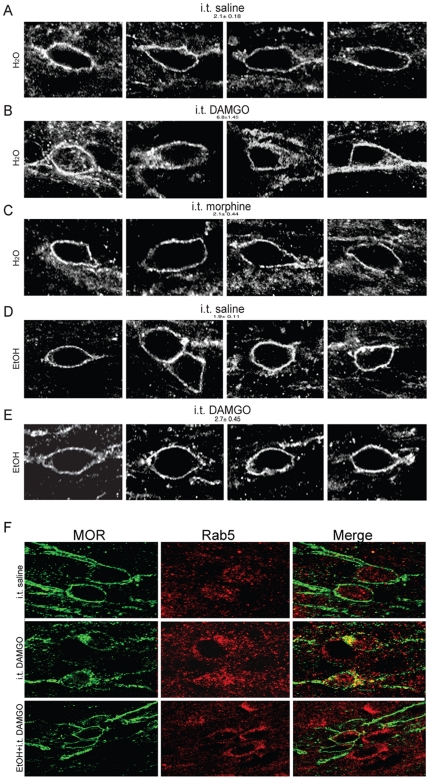
MOR distribution in lamina II neurons of the spinal cord after 8 weeks of drinking. (A–E) Distribution of the MOR. MORs were primarily localized to the plasma membrane of neurons after injection of saline in rats drinking either water (A) or ethanol (D). DAMGO (B), but not morphine (C), promoted endocytosis of the MOR in water drinking rats. (E) DAMGO did not promote endocytosis of MOR in ethanol drinking rats. The numbers over each panel represent the average number of intracellular vesicles per neuron (n>-90 neurons per group, 2–5 rats per group) p<0.001: i.t. DAMGO vs. all other groups (see Methods). (F) Co-immunostaining of MOR (green) and Rab5 (red) in water or ethanol drinking rats. MOR was co-localized with Rab5 after DAMGO treatment in water drinking rats, but not in ethanol drinking rats.

As mentioned above, we have previously observed that failure to endocytose the MOR can promote antinociceptive tolerance [35] and see Figure S1A). Thus, we investigated whether ethanol promoted antinociceptive tolerance to opioids by altering the ability of the MOR to endocytose in response to opioid peptide (Figure 3). As expected, DAMGO, but not morphine, administered i.t. promoted endocytosis of the MOR in the spinal cord of rats drinking only water (Figure 3B,C), consistent with previous findings [36], [37]. These endocytosed MORs co-localized with Rab5, a marker of early endosomes (Figure 3F). However, in the rats that had been drinking ethanol for 8 weeks, not only morphine (data not shown), but also DAMGO, failed to promote endocytosis of the MOR (Figure 3E), which was confirmed by a lack of MOR and Rab5 co-localization after DAMGO treatment in rats drinking ethanol. (Figure 3F, and see Figure S2 for additional z-stacks). Together, these results suggest that chronic ethanol drinking alters the ability of MOR to endocytose in response to opioid peptides.

### Chronic drinking causes downregulation of GRK2

**Figure 4:**
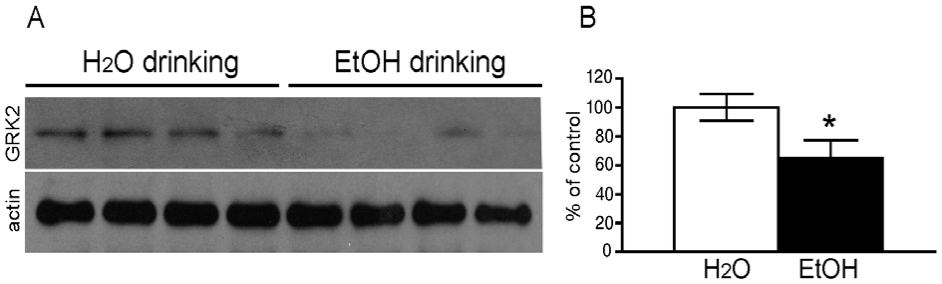
The effect of ethanol drinking on GRK2 protein levels in rat spinal cord. After 8 weeks of drinking, GRK2 protein levels were assessed by immunoblot in ethanol and water only drinking rats. Data are expressed as mean ± SEM. Tissue samples were analyzed from 4 rats in each group and three separate drinking experiments were conducted. * p<0.05, compared with the water drinking group.

We next examined the possible mechanisms by which chronic drinking caused decreased DAMGO-induced MOR endocytosis. Endocytosis of the MOR in response to agonist is initiated by phosphorylation of the receptor by members of the GRK family kinases. This is likely a rate limiting step, since over-expression of GRK, and in particular GRK2, can promote MOR endocytosis even to morphine [38], [39]. We found that the levels of GRK2 protein in the spinal cord of the rats who had been drinking ethanol for 8 weeks was significantly lower than that in the water drinking rats (Figure 4A, B). GRK2 levels were no different in ethanol and water drinking mice after only 4 weeks of drinking (Figure S3). These data suggest a mechanism by which chronic ethanol consumption can decrease endocytosis of the MOR.

### Chronic ethanol consumption reduces the function of MOR

**Figure 5:**
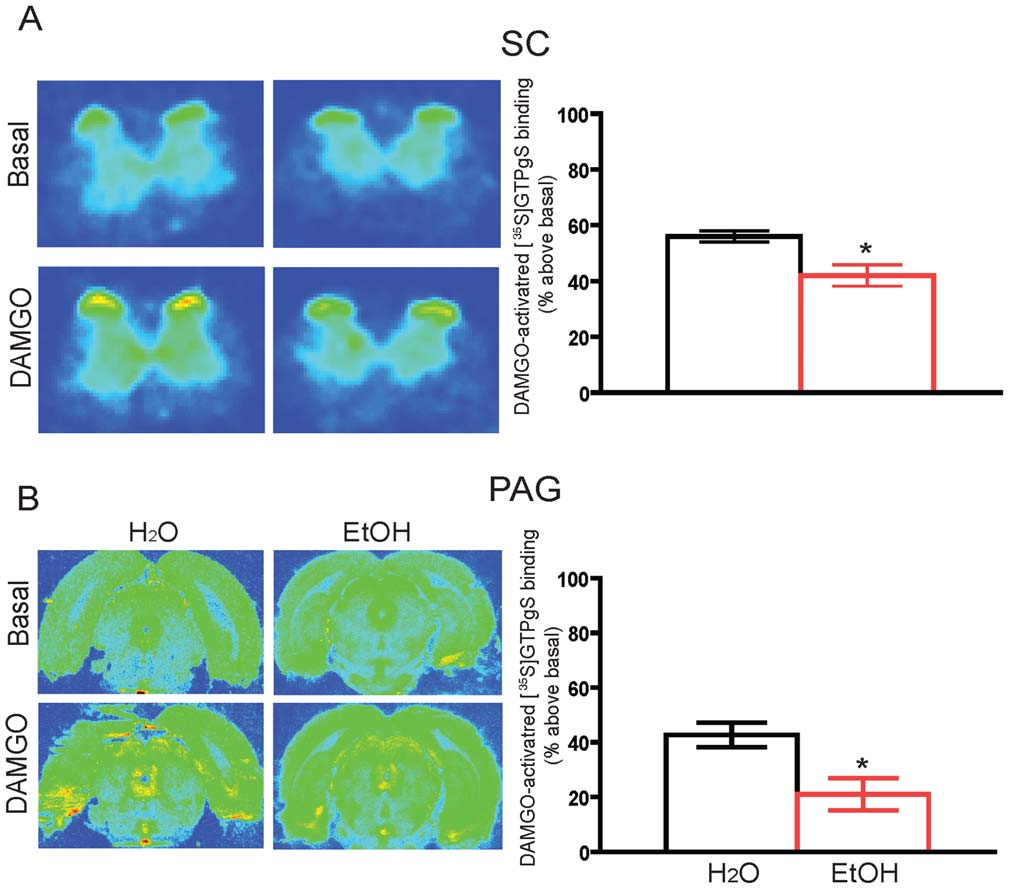
MOR signaling in spinal cord and PAG sections from water and ethanol drinking rats. After 8 weeks of drinking, there were significant decreases in DAMGO-mediated [^35^S]GTPγS binding in ethanol drinking compared to water drinking rats. Results are expressed as the mean ± SEM value from 3–4 rats in each group with 4–6 sections for each rat. * p<0.05, compared with water drinking group. SC  =  spinal cord; PAG  =  periaqueductal gray.

We next assessed the functional responsiveness of MOR to opioid in the spinal cord as well as several brain regions in rats that had been drinking water or ethanol. Importantly, DAMGO-activated [^35^S]GTPγS binding was significantly decreased in the spinal cord of ethanol drinking rats compared to water drinking rats after 8 weeks of drinking (Figure 5A), even though receptor number was not downregulated (Figure 2). In addition, a significant reduction in DAMGO-activated [^35^S]GTPγS binding was found in the periaqueductal grey (PAG) of ethanol drinking rats (Figure 5B). Both of these regions of the CNS are critical for nociception. No other brain regions examined showed significant changes in DAMGO-activated [^35^S]GTPγS binding (Figure S4A). In addition, there were no changes in DAMGO-activated [^35^S]GTPγS binding in spinal cord after only 4 weeks of drinking (Figure S4B).

## Discussion

Here we show that chronic ethanol consumption produces antinociceptive tolerance to exogenously administered opioids. We found that this tolerance was not accompanied by downregulation of MOR levels [40]. Rather, we found that chronic ethanol consumption causes a downregulation of GRK2 and thereby significantly impedes the ability of the enkephalin DAMGO to promote MOR endocytosis. Although many studies have clearly demonstrated that ethanol exerts at least some of its biological effects through the MOR, we believe that ours is the first study to show that voluntary ethanol drinking modulates MOR trafficking.

There is increasing evidence that receptor trafficking is a critical process for the modulation of signal transduction from the MOR. Ligands that promote endocytosis of the MOR have a reduced propensity to cause antinociceptive tolerance compared to morphine which does not [41]–[43] and see Figure S1A). In addition, pharmacological [35], [37], [44] or genetic [45] manipulations that allow morphine to promote endocytosis delay or prevent the development of tolerance. Importantly, here we show that chronic ethanol consumption changes trafficking of the MOR in response to DAMGO. The rats in our study are only exposed to DAMGO at two points: 4 weeks and 8 weeks. Hence, it is unlikely that antinociceptive tolerance was induced by failure of MOR to endocytose in response to DAMGO during these two exposures. Instead, we favor the hypothesis that chronic ethanol drinking has decreased the ability of the MOR to endocytose in response to the endogenous endorphin and enkephalin opioid peptides. These opioid peptides are released tonically in some circuits, but ethanol has been shown to cause a significant increase in opioid peptide concentration as well. Thus, we propose that chronic drinking prevents the ability of these opioid peptides to promote receptor endocytosis and, thereby, produces tolerance to opioids by mechanisms similar to those produced by morphine.

Multiple mechanisms could contribute to the ability of ethanol to prevent MOR endocytosis. Here we found that there was a significant decrease in GRK2 protein levels in the spinal cord of rats drinking ethanol for 8 weeks. While we can not rule out the possibility that this is a coincidence, it is intriguing that over-expression of GRK2 has been shown to facilitate morphine-induced MOR endocytosis, suggesting that GRK2 is a rate limiting step in MOR trafficking. In support of this hypothesis, previous studies have shown that disrupting GRK2 activity through over-expression of a dominant-negative mutant lacking kinase activity significantly reduced MOR endocytosis promoted by DAMGO [46] or etorphine [39] and consequently the functional affect of opioid drugs at the MOR [47]. Thus, while there are many ways MOR endocytosis could be adversely affected by ethanol, we favor the hypothesis that downregulation of key components of the endocytic machinery, or a loss of balance in components of this machinery could contribute to this decrease.

Failure to promote endocytosis could have varying functional effects on receptor-mediated signal transduction. Previous studies have found that blocking MOR endocytosis can significantly prolong the re-sensitization process for the receptor [48], and also promote enhanced receptor desensitization and antinociceptive tolerance to DAMGO [49]. To examine whether drinking promoted desensitization of the MOR, we examined the ability of the MOR to couple to G protein. Indeed, we found that MOR activity was significantly decreased in the ethanol drinking rats in two areas of the CNS important for opioid-induced antinociception: PAG and spinal cord (Figure 5A, B). Importantly, this reduction in activity was not accompanied by a reduction in MOR density (Figure 2). It is not clear why receptor activity would be more affected in spinal cord and PAG compared to other brain regions. However, chronic morphine treatment has been shown to selectively cause MOR desensitization in spinal cord and brain stem, but not other brain regions [50], [51]. Thus, our results are not only consistent with other reports [16]–[18], [52], but together our data suggest that the failure of the MOR to endocytose in response to activation after chronic ethanol drinking prevents the ability of the receptor to recover from desensitization and thereby promotes behavioral antinociceptive tolerance to opioid.

In summary, this study found that chronic alcohol intake significantly impedes the ability of opioid peptides to endocytose MOR, which leads to a decrease in the functional responsiveness of MOR and behavioral antinociceptive tolerance. These results suggest that chronic ethanol promotes adaptive changes in the opioid system that are presumably mediated solely by the presence of endogenous opioid. However, these results have important implications for alcoholics, especially since the primary therapeutic used in the treatment of alcoholism is naltrexone, an opioid receptor antagonist. Indeed, it will be important in future studies to examine whether similar changes in opioid receptor signaling occur in the brain after chronic ethanol consumption as we see in the spinal cord, since these changes this could effect responsiveness to naltrexone treatment. In addition, our studies suggest that higher doses of exogenous opioid drug would be necessary to treat pain in alcoholics and perhaps even heavy social drinkers.

## Supporting Information

Figure S1 **The antinociceptive effects of morphine (20 nmol/rat) or DAMGO (1 nmol/rat) following repeated intrathecal (i.t.) drug administration and the antinociceptive effect of i.t. morphine following ethanol consumption.** (A) Rats were injected i.t. with either morphine (20 nmol/rat) or DAMGO (1 nmol/rat) twice daily for 5 days and the antinociceptive effects of the drugs were measured on days 1 and 5. Rats developed significant antinociceptive tolerance to morphine, but not to DAMGO. (N =  6 for both morphine and DAMGO groups; *p< 0.05: day 5 vs. day 1) (B) Rats were tested for i.t. morphine antinociception 4 and 8 weeks after the beginning of ethanol drinking. The antinociceptive effect of morphine was significantly reduced after 8 weeks of ethanol drinking compared to water drinking group. (N = 6 for both water and ethanol drinking groups). *p< 0.05; EtOH vs. H_2_O drinking group).
(TIF)

Figure S2 **Immunohistochemical analysis of MOR distribution 1 µm z-sections.** Shown are 1 µm sections from top to bottom of individual spinal cord neurons for each treatment group. Pronounced MOR endocytosis was observed only in water-drinking rats treated with i.t. DAMGO but not in the other groups.
(TIF)

Figure S3 **The effects of 4-week ethanol drinking on GRK2 protein levels in rat spinal cord.** GRK2 protein levels were assessed by immunoblot in ethanol and water only drinking rats. Data are expressed as mean ± SEM. Tissue samples were analyzed from 3 rats in each group and 4 experiments were conducted. There was no statistically significant difference between the two groups.
(TIF)

Figure S4 **MOR signaling in several brain regions.** (A) No significant changes were observed in DAMGO-mediated [^35^S]GTPγS binding in multiple brain regions after 8 weeks of drinking in ethanol versus water drinking rats. (B) No significant changes were observed in DAMGO-mediated [^35^S]GTPγS binding in the spinal cord of between ethanol drinking and water drinking rats after only 4 weeks of drinking. Results are expressed as the mean ± SEM value from 3-4 rats in each group with 4-6 sections for each rat. Str =  striatum; NAc =  nucleus accumbens; Thal =  thalamus; Amyg =  amygdale.
(TIF)
