# Supplementary figures and images for: Chronic Ethanol Consumption in Rats Produces Opioid Antinociceptive Tolerance through Inhibition of Mu Opioid Receptor Endocytosis
Source: PLoS One. 2011 May 13;6(5):e19372. doi: 10.1371/journal.pone.0019372 (PMC3094338; doi:10.1371/journal.pone.0019372)

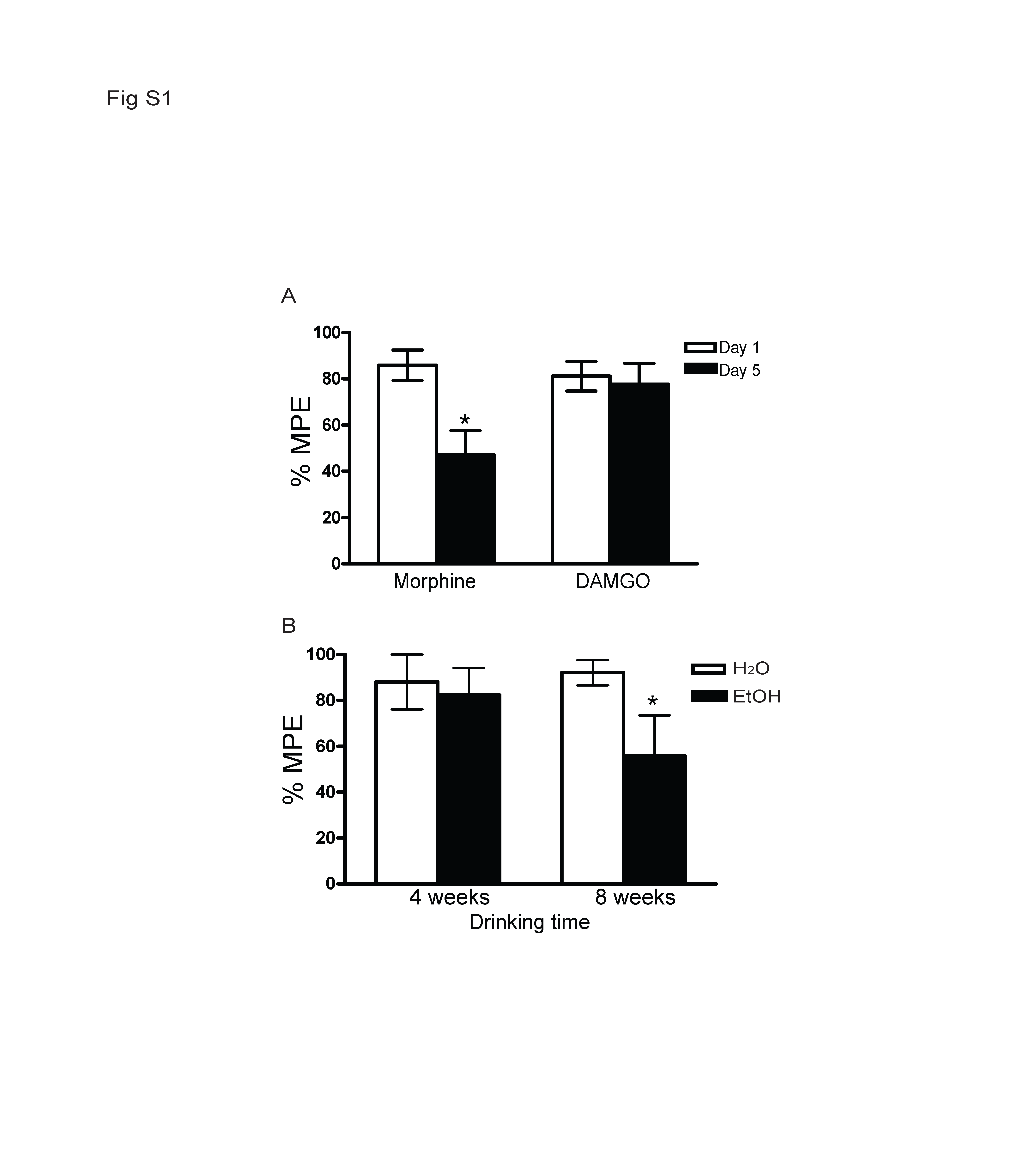

Supplement: Figure S1 — The antinociceptive effects of morphine (20 nmol/rat) or DAMGO (1 nmol/rat) following repeated intrathecal (i.t.) drug administration and the antinociceptive effect of i.t. morphine following ethanol consumption. (A) Rats were injected i.t. with either morphine (20 nmol/rat) or DAMGO (1 nmol/rat) twice daily for 5 days and the antinociceptive effects of the drugs were measured on days 1 and 5. Rats developed significant antinociceptive tolerance to morphine, but not to DAMGO. (N = 6 for both morphine and DAMGO groups; *p< 0.05: day 5 vs. day 1) (B) Rats were tested for i.t. morphine antinociception 4 and 8 weeks after the beginning of ethanol drinking. The antinociceptive effect of morphine was significantly reduced after 8 weeks of ethanol drinking compared to water drinking group. (N = 6 for both water and ethanol drinking groups). *p< 0.05; EtOH vs. H2O drinking group). (TIF) [file pone.0019372.s001.tif]

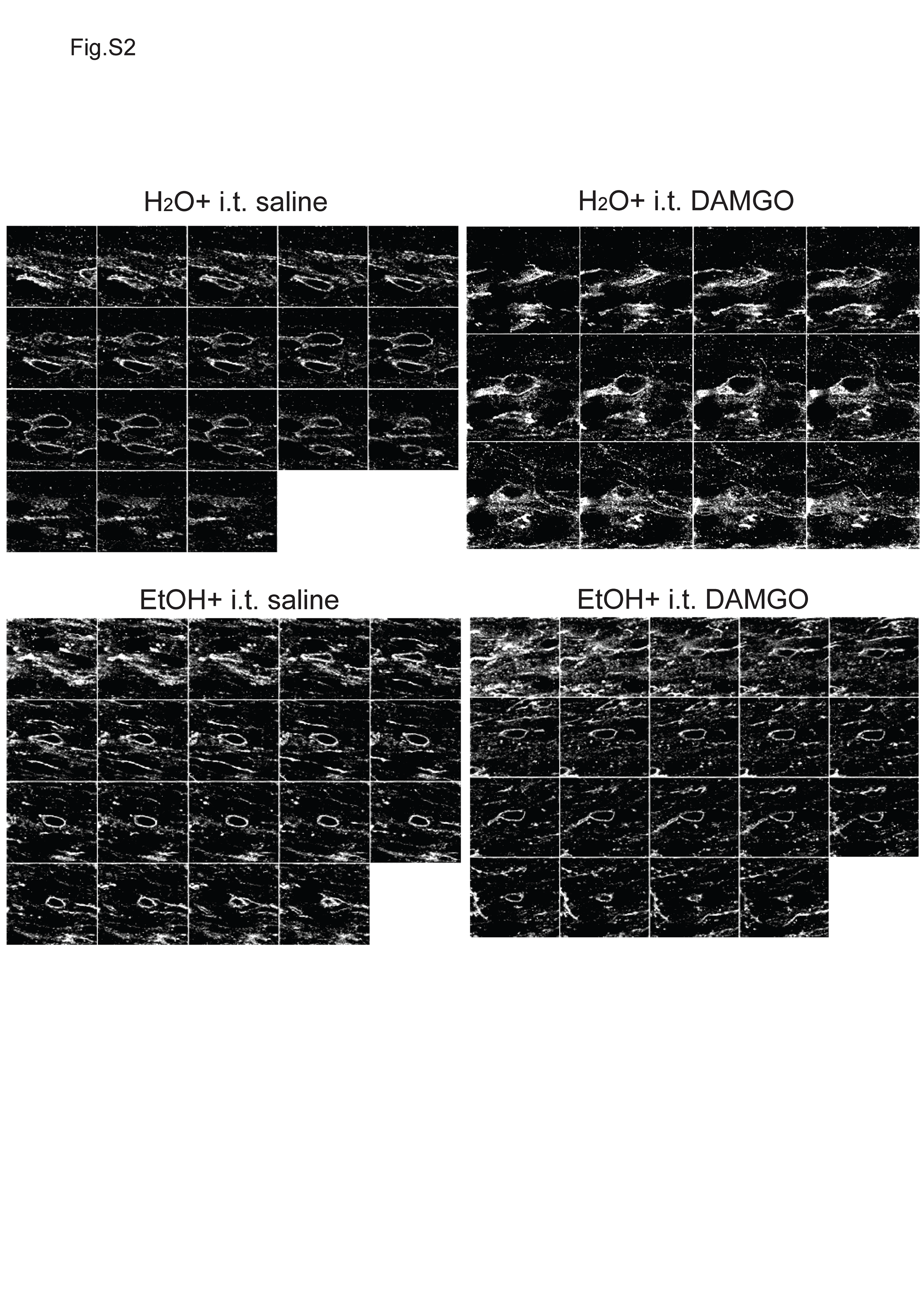

Supplement: Figure S2 — Immunohistochemical analysis of MOR distribution 1 µm z-sections. Shown are 1 µm sections from top to bottom of individual spinal cord neurons for each treatment group. Pronounced MOR endocytosis was observed only in water-drinking rats treated with i.t. DAMGO but not in the other groups. (TIF) [file pone.0019372.s002.tif]

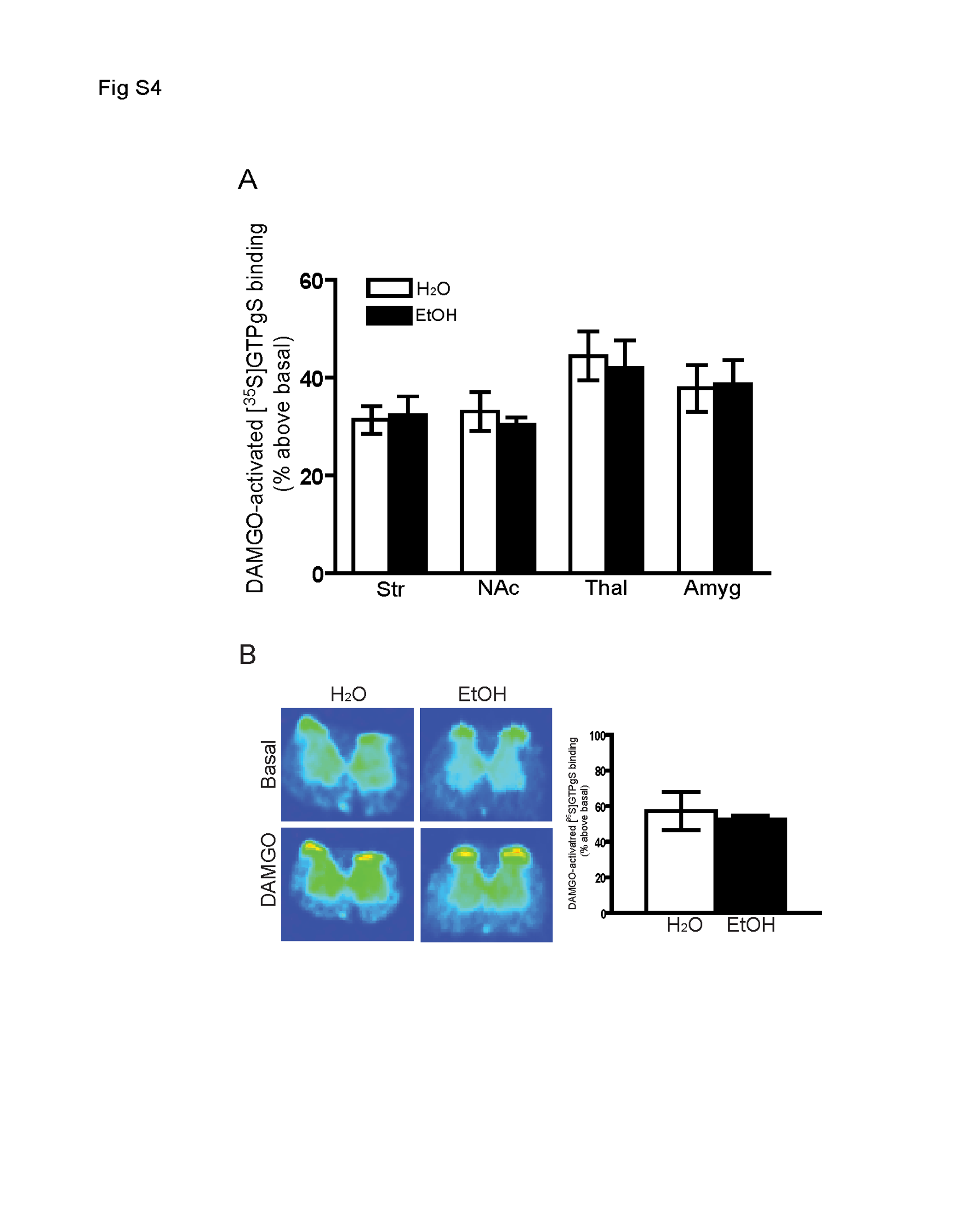

Supplement: Figure S4 — MOR signaling in several brain regions. (A) No significant changes were observed in DAMGO-mediated [35S]GTPγS binding in multiple brain regions after 8 weeks of drinking in ethanol versus water drinking rats. (B) No significant changes were observed in DAMGO-mediated [35S]GTPγS binding in the spinal cord of between ethanol drinking and water drinking rats after only 4 weeks of drinking. Results are expressed as the mean ± SEM value from 3-4 rats in each group with 4-6 sections for each rat. Str = striatum; NAc = nucleus accumbens; Thal = thalamus; Amyg = amygdale. (TIF) [file pone.0019372.s004.tif]
